# Abundance, characterization, and health risk evaluation of microplastics in borehole water in Birnin Kebbi, Nigeria

**DOI:** 10.5620/eaht.2024017

**Published:** 2024-06-07

**Authors:** Tajudeen Yahaya, Mutiyat Kehinde Adewale, Abdulgafar Bala Ibrahim, Baliqees Abdulkadir, Chikelu Chinelo Emmanuela, Adamu Zainab Fari, Asiya Koko Attahiru, Joseph Dahali Wanda

**Affiliations:** 1 Department of Biological Sciences, Federal University Birnin Kebbi, Kebbi State, Nigeria

**Keywords:** Fibers, Health hazards, Microplastics, Polymers, Polyethylene

## Abstract

Microplastic pollution has become a global menace, and water, being a major "sink" for pollutants, represents a significant source of human exposure. This study aimed to assess the safety of borehole water in Birnin Kebbi, Nigeria, specifically concerning microplastic pollution. Water samples were collected from boreholes in selected areas, including Bayan Kara, Malali, Rafin Atiku, Aliero Quarters, GwadanGaji, FUBK Takeoff Site, Kalgo Market, and Tarasa. Microplastics were extracted from the water samples through filtration using glass fiber filter papers, and were subsequently subjected to spectroscopy and microscopy to determine concentrations, shapes, and polymer types. Health risks associated with the microplastics were also calculated. The results revealed that the samples from Tarasa exhibited the highest concentrations of microplastics (96.967 particles/L), followed by Bayan Kara (92.70 particles/L), Rafin Atiku (92.33 particles/L), GwadanGwaji (92.30 particles/L), FUBK Takeoff Site (91.07 particles/L), Aliero Quarters (90.43 particles/L), Kalgo Market (88.00 particles/L), and Malali (86.40 particles/L). The most dominant shape was fibers (73 %), followed by fragments (16 %), foams (6 %), and filaments (5 %). Polyethylene and polyamide, in that order, were the most dominant polymers, while polystyrene was the least common. The majority of risk scores were classified as III. It can be inferred from the results that microplastic pollution in borehole water poses a health hazard in the city. Consumers of borehole water in the studied areas are advised to treat the water before consumption to mitigate potential health risks.

## Introduction

Microplastics, defined as plastics with a size of less than 5 mm, are frequently encountered in soil, water, the atmosphere, and living organisms. Comprising carbon and hydrogen atoms bound together in organic polymer chains [1], these polymers encompass polyethylene, polypropylene, polyvinyl chloride, and polyester, with polypropylene and polyethylene being the most predominant [2]. Microplastics are categorized as primary or secondary. Primary microplastics, known as microbeads, are microscopic (< 1 mm) and are manufactured for products like facial scrubs, exfoliators, cleansers, soaps, detergents, and plastic fibers used in synthetic textiles (e.g., nylon) [3,4]. Secondary microplastics result from the photo-degradation of larger plastics and are more prevalent than primary microplastics [5]. Photo-degradation of secondary microplastics is commonly facilitated by solar ultraviolet radiation, wind, currents, and other natural factors [6]. Microplastics exhibit diverse shapes or forms, including fiber, film, foam, spheres, and pellets [7].

Being non-biodegradable, microplastics persist and eventually transform into nanoplastics [8]. This property also enables them to traverse long distances in the environment, posing both health and environmental hazards. Although there is limited information on the health effects of microplastic exposures, studies suggest they can induce cellular and physiological changes in the body [9, 10]. Additionally, microplastics have been linked to obesity, cardiovascular disease, reproductive disorders, and breast cancer [9]. Laboratory studies have demonstrated significant cell damage and even cell death due to microplastic exposure [11]. Human exposure to microplastics is also believed to result in lower metabolic activities, DNA damage, oxidative stress, and inflammation [12]. The impacts of microplastics can be transmitted through various levels of the food chain, adversely affecting organisms [13]. The effects vary depending on the species of the organism and the type and concentration of microplastics [13].

Water, serving as a major "sink" for pollutants, represents a primary source of chronic exposure to microplastics. Exposure can occur through drinking, bathing, during the primary production of food, cleaning and sanitation of food processing plants, as an ingredient or component of food ingredients, and through various processing operations [14]. To minimize microplastic exposure and health risks, periodic monitoring of water sources is imperative. In Nigeria, groundwater (boreholes and wells) is the predominant source of drinking and domestic water due to its cost-effectiveness, availability, and easy maintenance. Additionally, there is a deficiency and inefficiency in piped water, mirroring the situation in developing nations. Given the threat posed by microplastic pollution through water, constant assessment of the pollution status of groundwater sources in the country is essential. Several studies, including those by Yalwajiet al. [15], Aliyu et al. [16], Attah et al. [17], and Ibetoet al. [18], have been conducted on microplastic pollution of water in Nigeria. However, literature searches revealed no studies in Kebbi State or its neighboring states. The majority of Kebbi residents depend on borehole water owing to the reasons mentioned earlier, yet they dump plastic materials indiscriminately within the metropolis, potentially entering groundwater sources, compromising their quality. Thus, evaluation of microplastic pollution status of groundwater in the city becomes necessary to raise public awareness. Therefore, this study aims to ascertain the abundance, polymer types, and health risks of microplastics in borehole water in Birnin Kebbi metropolis.

## Materials and Methods

### Description of the study site

**Figure 1.**
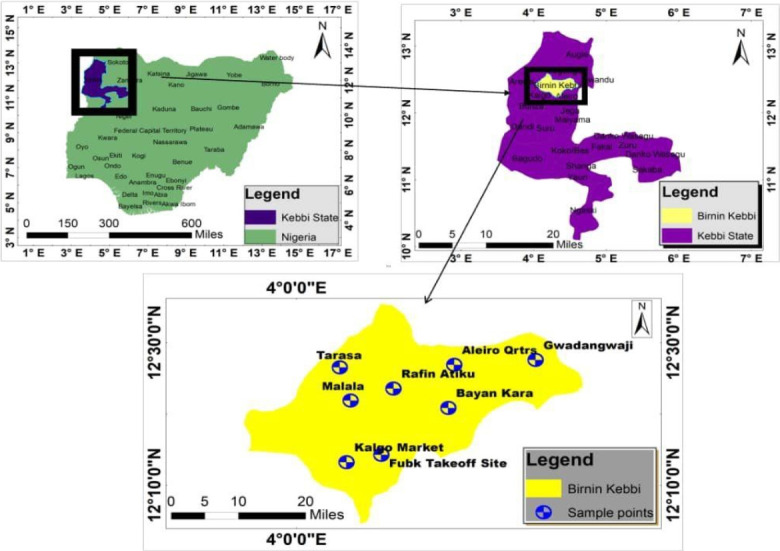
Map of the study area (drawn with ArcGIS version 10.3).

This study was conducted in Birnin Kebbi, situated in northwestern Nigeria. The city serves as both the capital of Kebbi State and the headquarters of the Gwandu Emirate. It is positioned along the Sokoto River at the crossroads of roads from Argungu, Jega, and Bunza. The geographical coordinates of Birnin Kebbi, Nigeria, are approximately 12° 27' 7.79" N latitude and 4° 12' 0.60" E longitude (Figure 1). Kebbi State, with a land area of 36,800 km2, has an estimated population of 4,531,129 based on 2006 projected census figures [19]. The major ethnic groups in the state include the Hausa, Fulani, Dakarki, and Kamberi, with Islam as the predominant religion. Notably, there are significant populations of settlers, mainly Yoruba, Igbo, and Nupe. The state experiences a semiarid climate, characterized by a savannah vegetation zone as part of the subSaharan Sudan belt in West Africa. The city's annual temperature averages 31.26 ℃ (1.8 % higher than Nigeria's averages), and it typically receives around 69.76 millimeters of precipitation with 94.53 rainy days annually (25.9 % of the time). Rainfall is concentrated in a brief wet season, extending from mid-May to early October (about 95 rainy days), while the dry season, with no precipitation, lasts for more than seven months.

Similar to other parts of the country, plastic materials, including plastic bags, polybags, sachet and bottled water, and jerrycans, are extensively used in the state. These materials are discarded in the environment and have the potential to enter groundwater sources, compromising their quality. This underscores the need for an evaluation of microplastic pollution in the city.

### Sample collection

Between August 2023 and early October 2023, triplicate borehole water samples were collected in pre-cleaned, non-plastic jars from various locations in Birnin Kebbi, Kebbi State, Nigeria, including Bayan Kara, Malali, Rafin Atiku, Aliero Quarters, GwadanGaji, FUBK Takeoff Site, Kalgo Market, and Tarasa areas. A total of twenty-four samples were collected and subsequently transported to the laboratory. The sampling was done during the rainy season when microplastic pollution is reported to peak [20].

### Microplastic extraction

The 24 water samples (3 from each location) underwent filtration using glass fiber filter papers, followed by digestion with 50 ml of hydrogen peroxide, and were agitated for a period of 5 days, as outlined by Wright et al. [21]. Subsequently, the digested materials were transferred into a separating funnel containing an aqueous potassium formate solution. In the funnel, particles in the water separated, with the finest particles settling in the lowest phase. The water in the lowest phase was then filtered using a nanopore inorganic membrane filter with a pore size of 0.2 µ m to isolate the microplastics. The filter was placed in Petri dishes, air-dried at room temperature, and stored in an airtight container to prevent contamination.

### Microplastic observation and identification

Petri dishes containing the filter were directly observed under a Nikon SMZ745 stereomicroscope (X40), where microplastics were counted and categorized as fibers, fragments, and films. Photographs of the particles were captured using a digital camera. The particles from each location were pooled together, after which identification, confirmation, and characterization of all the microplastics detected were conducted with a Nicolette Nexus 470 Attenuated Total Reflection ATR-FTIR by Therm, USA, utilizing Omic software. The spectral range for the spectrum in this study was 650-4000 cm^-1^, and the resolution was 4 cm^-1^. To correct for errors, a background air spectrum was frequently run throughout the process.

### Contamination control

All equipment and materials used were devoid of plastics and prewashed with ultrapure water, maintaining a clean laboratory environment. Throughout the analyses, plastic-free laboratory attire, including coats, gloves, and goggles, was worn. Each sample was meticulously labeled with pertinent information such as the date, time, GPS coordinates, and location.

### Microplastic risk assessment

The polymer risk index (PRI) was used to assess the risk of microplastics in the water samples, as shown in equation 1.


(1) PRI = ∑ PMP x PS

In equation 1, PMP represents the percentage of each microplastic polymer in the water samples, while PS denotes the score (constant) assigned to each polymer. The assigned scores are as follows: Polypropylene (PP) = 1, Polyethylene (PE) = 11, Ethylene Vinyl Acetate (EVA) = 22, Polystyrene (PS) = 30, and Polyamide (PA) = 47 [2]. These hazard scores were obtained from literature, which were ranked based on monomer classification, annual global waste generation, particle size, degradation time, and mean density of each polymer [22]. The Risk Level (PRI) was categorized based on the following scoring ranges: less than 10 was designated as Risk Level I, 10-100 as Risk Level II, 100-1000 as Risk Level III, and greater than 1000 as Risk Level IV [2].

### Data analysis

Microplastic abundance in the water samples was expressed as the number of microplastics per 1 liter of water (microplastic/L). All values were presented as mean ± standard deviation using Minitab 16.0. Minitab was also used to calculate the polymer risk index (PRI).

## Results

### Abundance and shapes of microplastics in the water samples

**Figure 2.**
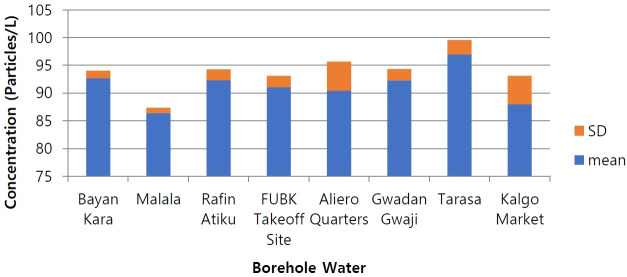
Concentrations of microplastics in the water samples

Figure 2 and Appendix 1 present the abundance of microplastics in the borehole water obtained from Birnin Kebbi. Among the water samples, Tarasa exhibited the highest concentration of microplastics (96.967 particles/L), followed by Bayan Kara (92.70 particles/L), Rafin Atiku (92.33 particles/L), GwadanGwaji (92.30 particles/L), FUBK Takeoff Site (91.07 particles/L), Aliero Quarters (90.43 particles/L), Kalgo Market (88.00 particles/L), and Malala (86.40 particles/L). The P-value is very low (less than 0.05), indicating that the null hypothesis can be rejected. This suggests that there is a statistically significant difference between the means of at least two of the eight samples. The GPS coordinates of the locations of the selected sample sites are shown in Table 1.

**Figure 3.**
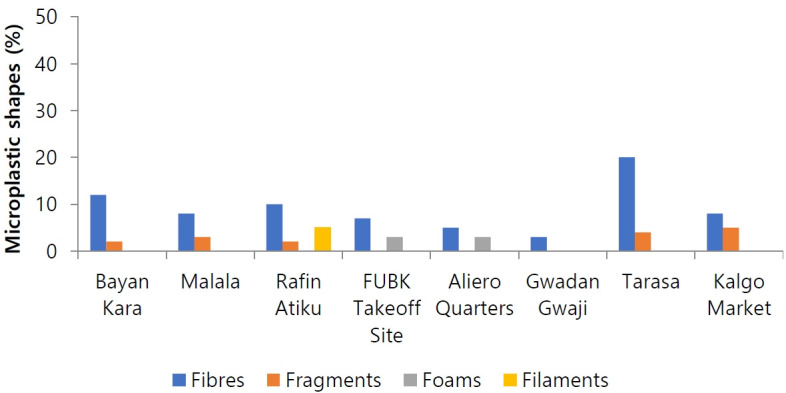
Percentage of microplastic shapes in the water samples.

**Figure 4.**
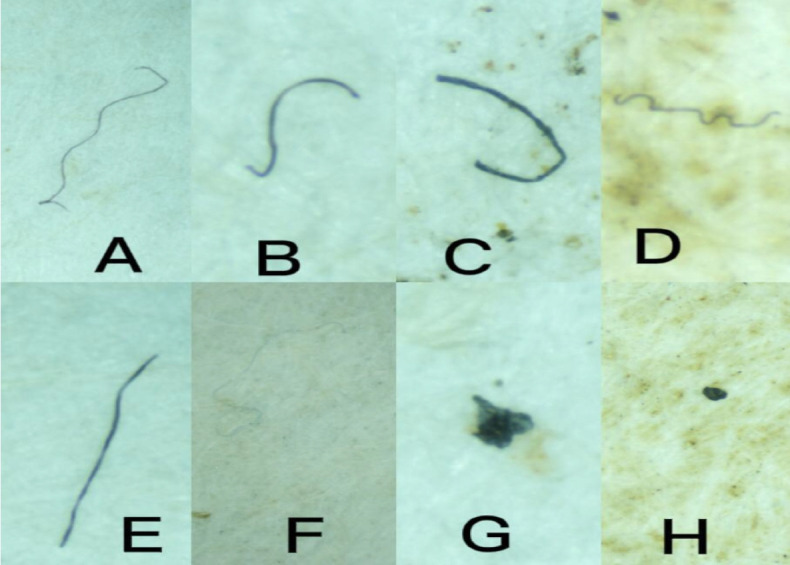
Microplastics shapes identified in the water samples: A, B, C, D and E are Fibers; F indicates filament; G represents fragments; and H represents foam

The percentage distribution of microplastic shapes in the water samples is detailed in Figure 3. Fibers comprised the highest percentage (73 %), followed by fragments (16 %), foams (6 %), and filaments (5 %). This suggests that fibers, fragments, foams, and filaments, in that order, were the predominant shapes in the borehole water samples. Photomicrographs depicting these microplastics are provided in Figure 4.

### Polymer type distribution in the water samples

Table 2 presents the spectral and functional groups of polymers in the water samples. The samples collected from Bayan Kara exhibit a stretching vibration at 2922.23 cm^-1^, typical of the -CH alkane group, suggesting polyethylene. Additionally, there is an absorption peak at around 2892.41 cm^-1^, characteristic of CH2, indicating polyamide. Another absorption at 2922.23 cm^-1^ is attributable to the asymmetrical C-H bending region, indicating polypropylene. Furthermore, there is an absorption peak at 1367.93 cm^-1^ synonymous with CH symmetrical stretching, suggesting polystyrene. An intense peak at 2068.00 cm^-1^ represents the C-H stretching group, suggesting ethylene vinyl acetate.

The samples from Malala exhibit an absorption peak of 2538.31 cm^-1^due to OH stretching, suggesting polyamide. There is also a 1237.47 cm^-1^ peak synonymous with CH3 symmetrical stretching, indicating polypropylene. Additionally, a vibration peak at 2236.04 cm^-1^, typical of C-H asymmetrical stretching, indicates ethylene vinyl acetate, and a 1364.20 cm^-1^ absorption characterizes C-H bending vibration, suggesting polystyrene.

The samples from Rafin Atiku contain an absorption peak at around 2899.86 cm^-1^, typical of the amine group, indicating polyamide. Another absorption peak at 2892.4 cm^-1^, characteristic of C-H asymmetrical stretching vibration, indicates polypropylene. There is also an absorption peak of 2173.03 cm^-1^ synonymous with the stretching region of the CH2 group, indicating ethylene vinyl acetate.

The samples obtained from FUBK Take-off Site have an absorption peak of 1051.10 cm^-1^, typical of CH3 symmetrical stretching vibration, indicating polypropylene. A peak at 3384.42 cm^-1^, characteristic of bending C-C alkane group, indicates polyamide. Additionally, a peak at 2042.58 cm^-1^, synonymous with C-H stretching, indicates ethylene vinyl acetate.

The samples from Aliero Quarters contain a peak at 2892.4 cm^-1^, typical of the CH2 group, indicating polyethylene. Additionally, a sharp peak at 3428.69 cm^-1^ is attributable to C-H, indicating polyamide.

The samples obtained from GwadanGaji contain an absorption peak at around 2899.86 cm^-1^ due to the bending vibration of the -CH2 group, indicating polyethylene. A peak at 894.56 cm^-1^ is attributable to C-H stretching, indicating polypropylene. Furthermore, a peak at 1601.7 cm^-1^ is typical of the vibration of C-C stretching, indicating polystyrene, and a characteristic peak of 2102.21 cm^-1^ is attributable to the C=O stretching of the carboxylic acid of the vinyl acetate group, indicating ethylene vinyl acetate.

The samples from Tarasa exhibit a peak at 2922.23 cm^-1^, accounting for C-H rocking vibration, indicating polyethylene. A peak at 1237.47 cm^-1^ represents C-H symmetrical stretching, indicating polypropylene. Additionally, a peak at 2538.31 cm^-1^ is attributable to stretching at the N=C=O isocyanate group, indicating polyamide.

The samples obtained from Kalgo Market contain a characteristic peak at 1934.48 cm^-1^, typical of C-H asymmetrical stretching, indicating polyethylene. A peak at 894.56 cm^-1^, synonymous with CH2, indicates polypropylene, and a peak at 1364.20 cm^-1^ is assigned to C-C stretching, indicating polystyrene.

**Figure 5.**
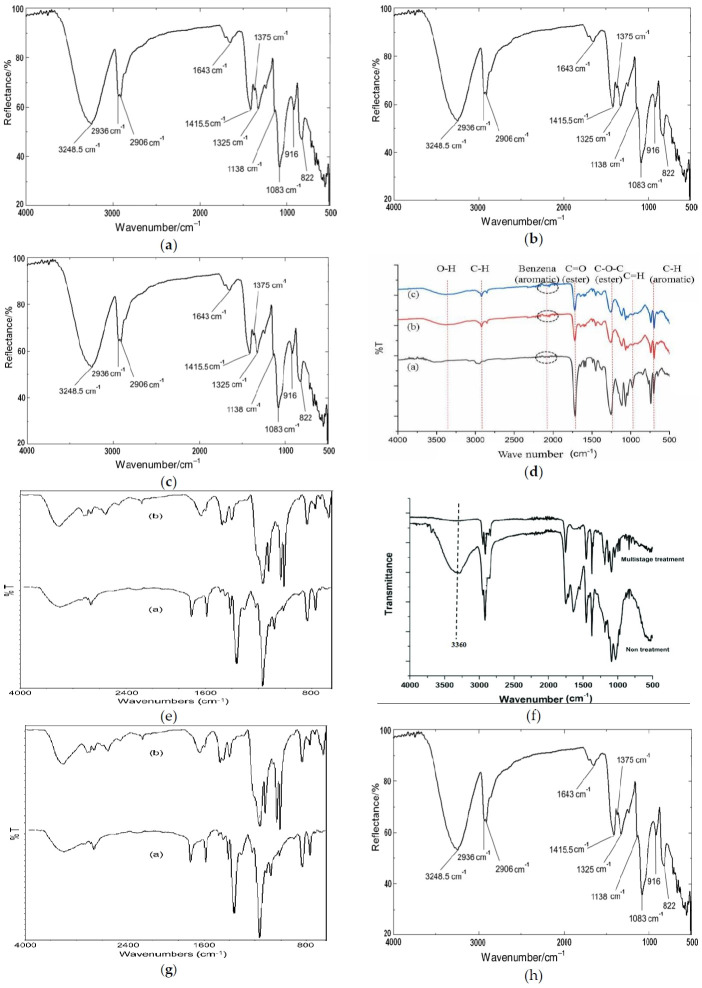
Spectra of the polymers in the water samples as obtained by FTIR: a = Bayan Kara, b = Malala, c = Rafin Atiku, d = FUBK Take-off Site, e = Aliero Quarters, f = GwadanGaji, g = Tarasa, h = Kalgo Market.

Figure 5 depicts the spectra of the functional groups of polymers identified in the water samples.

**Figure 6.**
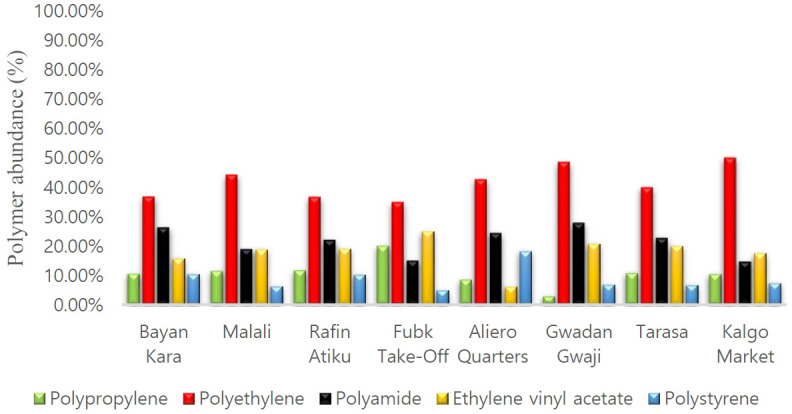
Polymer types in the water samples

Figure 6 depicts the percentage distributions of polymer types in the water samples. Polyethylene emerged as the dominant polymer, constituting 50.00 % in Kalgo Market, 48.60 % in GwadanGwaji, 44.30 % in Malali, 42.70 % in Aliero Quarters, 40.00 % in Tarasa, 36.80 % in Bayan Kara, 36.70% in Rafin Atiku, and 35.00 % in FUBK Takeoff Site. Following closely is polyamide, with percentages of 27.80 % in GwadanGwaji, 26.30 % in Bayan Kara, 24.40 % in Aliero Quarters, 22.70 % in Tarasa, 22.10 % in Rafin Atiku, 18.90 % in Malali, 15.00 % in FUBK Takeoff Site, and 14.70 % in Kalgo Market. Ethylene vinyl acetate accounted for 25.00 % in FUBK Takeoff Site, 20.80 % in GwadanGwaji, 20.00 % in Tarasa, 19.10 % in Rafin Atiku, 18.90 % in Malali, 17.60 % in Kalgo Market, 15.80 % in Bayan Kara, and 6.10 % in Aliero Quarters. Polypropylene made up 20.00 % in FUBK Takeoff Site, 11.70 % in Rafin Atiku, 11.40 % in Malali, 10.70 % in Tarasa, 10.50 % in Bayan Kara, 10.30 % in Kalgo Market, 8.50 % in Aliero Quarters, and 2.80 % in GwadanGwaji. Polystyrene accounted for 18.30 % in Aliero Quarters, 10.50 % in Bayan Kara, 10.30 % in Rafin Atiku, 7.40 % in Kalgo Market, 6.90 % in GwadanGwaji, 6.70 % in Tarasa, 6.30 % in Malai, and 5.00 % in FUBK Takeoff Site.

### Polymer risk index of the water samples

Table 3 presents the risk index of the polymers in the water samples. Polystyrene, ethylene vinyl acetate, and polyethylene, across all locations, registered a risk level III. Polypropylene exhibited a risk level II, and polyamide demonstrated a risk level IV.

## Discussion

The present study aimed to assess the abundance, distribution, and health risks associated with microplastics in borehole water in Birnin Kebbi, Nigeria. Microplastics were identified in all water samples from the boreholes, ranging from 86 to 97 particles/L, which is lower than the range of 206 to 1691 particles/L found in borehole water in Lagos by Oni and Sanni [28]. It is also lower than the 153 particles/L detected by Aliyu et al. [16] in untreated water in Kaduna, Nigeria, but higher than the 10 to 34 particles/L reported in borehole water in Northwest Mexico by Alvarado-Zambrano et al. [29]. Similarly, it exceeds the 5.00 to 10.5 particles/L found in water in China by Chen et al. [30]. Oni and Sanni [28] proposed that locations with elevated microplastic levels might be attributed to high population density and intense industrial activities, while areas with lower microplastic abundance could result from lower population densities and reduced industrial activities. Similarly, Jessieleenaet al. [31] suggested that municipal wastewater, dependent on population and anthropogenic activities, is a major contributor to microplastics in aquatic environments. In contrast to other cities in Nigeria mentioned above, Birnin Kebbi is less populated and industrialized, potentially explaining its lower pollution levels. Advanced cities mentioned above adhere to strict environmental protection laws, preventing indiscriminate waste dumping, including plastic materials, which may contribute to their less polluted groundwater despite industrialization and higher population density compared to Birnin Kebbi.

Figure 3 illustrates the percentage distribution of individual microplastic shapes in the water samples. Across all locations, fibers were the predominant shape, followed by fragments, foams, and filaments. This aligns with findings by Olarinmoyeet al. [32] and Yahaya et al. [2] in Lagos and Badagry Lagoons, respectively, where fibers dominated water samples. Inconsistently, Ebere et al. [33] and Oni and Sanni [28] reported fragment dominance in water in Imo State and Lagos State, respectively. Aliyu et al. [16] found a prevalence of fragments in water in Kaduna. Microplastic fibers, often used in clothing and detergents, may originate from clothing and laundry, especially wastewater discharged from manual washing and washing machines [34]. Hard plastics such as bottles, kegs, buckets, and outer packaging, visible in the studied area, may be sources of fragments, while plastic bags like poly bags and food packaging could contribute to foams [35].

Figure 6 depicts the percentage distribution of polymer types in the water samples, revealing polyethylene as the most abundant polymer in all locations, followed by polyamide, except in Rafin Atiku and Kalgo Market, where ethylene vinyl acetate exceeded polyamide. In Lagos, Fred-Ahmadu et al. [36] identified polyethylene and polypropylene as the most abundant polymers. Oni and Sanni [28] reported polypropylene and polyethylene dominance, consistent with Aliyu et al. [16] in Kaduna. Notably, polyamide's prevalence in drinking water in Birnin Kebbi is a unique finding in Nigeria. Polyethylene's ubiquity is attributed to its use in daily materials such as plastic bags, films, containers, and bottles. Polypropylene, used in textiles and durable materials, and polyamide, primarily for nylon production, serve various applications [37].

Table 3 displays the risk levels of various polymers in borehole water samples, calculated using polymer risk index, a mathematical model. Polystyrene, ethylene vinyl acetate, and polyethylene exhibited risk level III across all locations, while polypropylene showed risk level II, and polyamide showed risk level IV. This indicates a prevailing moderate risk (level III), with polyamide posing the most significant risks. These findings suggest potential health hazards for consumers. Studies link polypropylene exposure to metastatic features in human breast cancer and colonic apoptosis, while polyethylene exposure promotes inflammation, organ damage, and chemical alteration of plastics already in the body. Polyamide ingestion induces lipid metabolism and intestinal disorders, while polystyrene ingestion inhibits reproduction and causes DNA damage. Overall, microplastic exposure induces various toxic effects, including oxidative stress, metabolic disorders, immune responses, neurotoxicity, and reproductive and developmental toxicity [38].

## Conclusions

Based on the results, it can be concluded that borehole water contains a significant amount of microplastics. The three predominant shapes observed among these microplastics are fibers, fragments, filaments, and foams, with fibers being the most prevalent. The two most common polymer types found in the borehole water at all sample points were polyethylene and polyamide, while polystyrene was the least frequently identified. The predominant risk score for these polymers is III, suggesting that the microplastics in the boreholes may pose some health and environmental risks. Notably, polyamide presents the highest risk, registering a risk score of IV. While we recommend further studies to substantiate our claims, consumers of groundwater in the affected areas should consider treating the water before consumption. Discouraging the indiscriminate dumping of plastic waste into the environment is crucial, and prioritizing the recovery and recycling of plastic will help reduce the environmental impact of plastic pollution.

## Declaration of generative AI and AI-assisted technologies in the writing process

During the preparation of this work the authors used ChatGPT in order to correct grammatical errors and make sentences flow. After using this tool, the authors reviewed and edited the content as needed and take full responsibility for the content of the publication.

## Figures and Tables

**Table 1.** GPS coordinates of the sampling sites.

Location	Latitude	Longitude
Bayan Kara	N 12°28096	E 4°11'6159
Malala	N 12°345423	E 4°20'7977
Rafin Atiku	N 12°26460	E 4°11'3527
FUBK Takeoff Site	N 12°20504	E 4°12'1388
Aliero Quarters	N 12°28272	E 4°15'2600
GwadanGwaji	N 12°28411	E 4°15'1032
Tarasa	N 12°28113	E 4°10'1316
Kalgo Market	N 12°345468	E 4°20'8014

**Table 2.** Absorbance peak and functional groups of polymers identified in the water samples.

Sample	Absorbance peak	Functional group	Polymer type	Confirmed from
Bayan Kara	2922.23, 2892.4, 1367.93, 2068.00 and 2892.41	C-H Stretch, CH2, C-H Bend, C-C bend alkane	PE, PA, PP, PS, EVA	[23]
Malala	2538.31, 1237.47, 2236.04 and 1364.20	C-H, CH3, O-H Stretch	PA, PP, EVA, PS	[24]
Rafin Atiku	2899.86, 2892.4 and 2173.03	CO-NH, C-H Stretch, CH2	PA, PP, EVA	[24]
FUBK Takeoff Site	1051.10, 3384.42, and 2042.58	CH3, C-H Stretch, C-C bend	PP, PA, EVA	[24]
Aliero Quarters	1237.47 and 3428.69	C-H, CH2	PE, PA	[24]
Gwadan Gaji	2899.86, 894.56, 1601.7 and 2102.21	CH2, C-H Stretch C=O, carboxylic acid group	PE, PP, PS, EVA	[25]
Tarasa	2922.23, 1237.47 and 2538.31	N=C=O, C-H Stretch	PE, PP, PA	[26]
Kalgo Market	1934.48, 894.56 and 1364.20	C-H Stretch, C-C Stretch	PE, PP, PS	[27]

**Table 3.** Risk index of polymers in the water samples.

Location	Polypropylene	Polyethylene	Polystyrene	Ethylene vinyl acetate	Polyamide
Bayan Kara	10.5	404.8	315.0	347.6	1236.1
Malali	11.4	487.3	189.1	415.8	1088.3
Rafin Atiku	11.7	403.7	309.0	420.2	1038.7
FUBK Takeoff	20.0	385.0	150.0	550.0	1105.0
Aliero Quarters	10.5	469.7	549.0	134.2	1146.8
GwadanGwaji	10.1	534.6	207.0	457.6	1306.6
Tarasa	10.7	440.0	201.0	440.0	1066.9
Kalgo Market	10.3	550.0	222.0	387.2	1001.9
Risk level	II	III	III	III	IV

Note: I = very low risk, II = low risk, III = moderate, IV = high, and V = very high [2]ables may have a footnote.

## Supplementary Material

The raw data on all the participants is available online at www.eaht.org.
